# The Consequences of Training and Competition to the Musculoskeletal System in Ultramarathon Runners: A Narrative Review

**DOI:** 10.3389/fphys.2021.738665

**Published:** 2021-09-24

**Authors:** Alicja Partyka, Zbigniew Waśkiewicz

**Affiliations:** ^1^Poznan University of Medical Sciences, Poznan΄, Poland; ^2^ Institute of Sport Science, Jerzy Kukuczka Academy of Physical Education, Katowice, Poland; ^3^ Department of Sports Medicine and Medical Rehabilitation, Sechenov First Moscow State Medical University, Moscow, Russia

**Keywords:** ultramarathon, musculoskeletal system, endurance sport, running, injury, muscle injury

## Abstract

Ultramarathons are becoming increasingly popular every year, leading to more and more publications focusing on athletes of these endurance events. This paper summarizes the current state of knowledge on the effects of ultramarathons on the motor system. Various studies have attempted to answer questions about negative and positive effects on the musculoskeletal system, common injuries, optimal strategies, and regeneration. Considering the increasing number of ultramarathon athletes, the discoveries may have practical applications for a multitude of experts in the field of sports medicine, as well as for the athletes themselves. Acute locomotor system changes in runners as assessed by locomotor biomarkers are reversible and may be asymptomatic or painful. Injuries suffered by runners largely allow them to finish the competition and are usually overlooked. Regeneration, including regular massage and the use of supporting techniques, allows for faster convalescence. This publication is meant to be a source of knowledge for people associated with this discipline.

## Introduction

Ultramarathon racing has been gaining followers in recent years and its popularity has grown significantly. The sport has seen an increase in the number of competitors as well as the number of organized competitions at distances above the marathon (Kim et al., 2007; Millet et al., 2011, 2012; Krabak et al., 2013; Kupchak et al., 2014; Thompson, 2017). Ultramarathon runners are a very specific group that differs considerably from other runners (Knechtle et al., 2012b; Freund et al., 2013). These athletes are highly motivated in terms of pursuing their passion. Although, they show above-average health awareness, information about the potential detrimental effects of running on their health does not stop most of them from continuing their training (Hoffman and Krouse, 2018).

Due to the specific differences of this discipline over traditional marathon running, research that delves into the characteristics of long-term training, competitions, and effects on ultramarathoners has become important (Hoffman and Krouse, 2018). In the field of research on ultramarathons, scientific investigation is still needed, as well as counseling within the scope of training and nutrition plans (Millet et al., 2011; Krabak et al., 2013; Thompson, 2017).

The literature emphasizes that, in ultramarathon running, one should use not only the advice addressed to marathon runners (Knechtle, 2012). The characteristics of runners who run marathons and those who run longer distances vary noticeably, and most notably in terms of training sessions (Khodaee and Ansari, 2012). In addition, little research has been conducted on the long-term effects of practicing this sport (Hoffman, 2016).

Although, limited work has focused on the musculoskeletal system of ultramarathon runners, the existing reports appear relatively consistent. Current literature emphasizes that the maximal oxygen consumption, VO_2_ max, the ability to generate energy, and the capacity to overcome mental resistance and fatigue are of key importance for the effective and efficient functioning of the ultramarathon runner’s body (Millet, 2011; Thompson, 2017). The musculoskeletal system of ultramarathon runners is subject to enormous loads, which leads to specific adaptations for this discipline (Crenshaw et al., 1991; Harber and Trappe, 2008; Knechtle et al., 2012b). The runner’s locomotor system, and its muscles in particular, is changed gradually, both at the cellular and tissue levels (Crenshaw et al., 1991; Harber and Trappe, 2008; Knechtle et al., 2012b; Ramos-Campo et al., 2016). On the other hand, training and competition strain frequently lead to injuries. Ultramarathoners deal with specific types of problems, of which those affecting the musculoskeletal system are the second most common (Khodaee and Ansari, 2012; Krabak et al., 2013, 2014; Hoffman and Krishnan, 2014; Hoffman, 2016; Vernillo et al., 2016).

In relation to the above considerations, the authors of this study attempted to characterize the impact of training and ultramarathon competitions on the movement systems of athletes. Methodical analysis of existing scientific reports will enable a more comprehensive characterization of the impact of loads on athletes and at the same time may become a guide for future scientific research by determining poorly understood areas. This work may also be a compendium of knowledge that allows athletes, coaches, doctors, and physiotherapists to increase awareness of the impact of ultramarathon training and the dangers it carries for the musculoskeletal system. For this purpose, an electronic literature search was conducted using PubMed database until October 2020. The following keywords were used: “ultra-marathon” or “ultramarathon.” We identified more than 700 studies meeting our initial search criteria. Articles that described footraces performed on the ultradistance were considered eligible rather than ultra-endurance exercise in general. Similarly, studies that examined aspects of musculoskeletal system in context of ultramarathon were included in this review. The reference lists of the articles meeting our inclusion criteria were searched for additional literature.

## Biomarkers of the Locomotor System

An organism subjected to an endurance effort, such as running an ultramarathon, responds to the stress through various bodily reactions that are reflected in laboratory indicators (Khodaee et al., 2015). Most often, these changes are caused by direct organ damage (Ramos-Campo et al., 2016; Martínez-Navarro et al., 2019). However, many factors influence changes in these biomarkers, starting from individual differences to the duration and conditions in which the run is performed (Harber and Trappe, 2008; Hoffman, 2016; Hoffman et al., 2016). In addition, reference values may not provide an accurate reflection of the duress experienced under these conditions, making interpretation of certain parameters difficult. In general, changes in the level of biomarkers of muscle and cartilage damage may suggest a detrimental effect of ultramarathon distances on the musculoskeletal system, leading to degenerative changes in the future (Chilibeck et al., 1995).

### Muscles

Many studies have drawn attention to the fact that completing an ultramarathon is associated with damage to muscle fibers (Kim et al., 2007, 2009; Millet et al., 2011; Krabak et al., 2014; Kupchak et al., 2014; Carmona et al., 2015; Khodaee et al., 2015; Degache et al., 2016; Hoffman et al., 2017). As with any physical activity, running also causes micro-injuries to muscle tissue, and various factors alleviate or aggravate these damages. The breakdown (catabolism) of striated muscle fibers after physical activity is a condition triggered by exercise of high total intensity (Khodaee and Ansari, 2012; Kerschan-Schindl et al., 2015; Larson et al., 2016). Eccentric contractions, which are more common in mountain ultramarathon runners, are particularly severe for myocytes. This is due to the fact that running downhill causes the muscle attachments to move apart under the influence of external force (Millet et al., 2011; Hoffman et al., 2012; Krabak et al., 2014; Carmona et al., 2015; Degache et al., 2016; Magrini et al., 2017). In the measurement of muscle mass, a noticeable loss occurs after completing the run (Knechtle et al., 2011, 2012a). The consequence of muscle microtrauma is the release of proteins such as creatine kinase (CK) into the bloodstream. CK level is widely believed to be a significant indicator of muscle injury. However, it is not fully specific for striated skeletal muscles (Fallon, 2001; Nieman et al., 2005; Kim et al., 2007; Hoffman et al., 2012; Khodaee and Ansari, 2012; Carmona et al., 2015; Magrini et al., 2017). The increase in CK depends on the degree of damage to the permeability of myocyte membranes. The level of CK increases with the extension of the ultramarathon distance (Kim et al., 2009; Carmona et al., 2015) and much more in its second half (Kim et al., 2007; Jastrzȩbski et al., 2015b; Son et al., 2015). Regardless, a large increase in CK due to ultramarathon running in most cases is asymptomatic and does not require hospitalization, unless there are other damaging factors (Skenderi et al., 2006; Millet et al., 2011; Kupchak et al., 2014; Magrini et al., 2017). Upon separation into its isoenzymes, the greatest increase is observed for the skeletal muscle fraction (creatine kinase isoenzyme MM, CK-MM), and a small but also noticeable increase is noted for the myocardial fraction (creatine kinase isoenzyme MB, CK-MB; Son et al., 2015). The level of the brain fraction (CK-BB) remains undetectable in these measurements. The degree of CK increase varies among athletes despite covering the same distance, an effect caused by a multitude of factors. However, training experience, years of participation in ultramarathon distances, and the highest weekly mileage are not correlated with plasma CK levels (Hoffman et al., 2012). Only one study found a difference in CK levels between athletes of different ages. The study examined CK levels after athletes covered 25, 50, and 75 out of 100km. Two age groups were distinguished: the older group (50.56±9.7years) showed a greater increase in CK level compared to the younger group (32±5.33years; Jastrzȩbski et al., 2015b). According to some authors, the correlation between finishing time and CK levels is present (161km; Hoffman et al., 2017; 100km; Jastrzȩbski et al., 2015b), and according to others, not (100km; Knechtle et al., 2011; 160km; Nieman et al., 2005; 161km skyrunning; Magrini et al., 2017; 161km; Hoffman et al., 2012; 246km; Skenderi et al., 2006). The leading ultramarathon runners show lower initial CK values than non-leaders, and also show lower values after finishing the race (Suzuki, 2002). However, CK level is correlated with delayed-onset muscle soreness (DOMS; Nieman et al., 2005; Francisco, 2013). Runners rated pain on a 10-point Likert scale as 7.1±0.3 at 1day after the run and as 5.0±0.3, 2.5±0.2, and 1.6±0.1 at 3, 5, and 7days after the run, respectively (Nieman et al., 2005). The levels were the highest immediately after the run and returned to the values from before the run within 1week (Nieman et al., 2005; Millet et al., 2011) nevertheless in 5days after the finish they were definitely close to the initial values (Kim et al., 2009; Hoffman et al., 2017). Soreness, soreness of the lower body, and general muscle fatigue disappear at the same rate, regardless of the CK level (Hoffman et al., 2017).

Activities such as ultramarathon running can also increase levels of the heart muscle marker troponin (Carmona et al., 2015; Khodaee et al., 2015; Zebrowska et al., 2020). Unlike individuals who have suffered myocardial infarction, where cardiac troponin I (cTnI) values increase and last up to 5days, ultramarathoners have lower cTnI values and return to normal after 24–48h (Carmona et al., 2015).

With regard to electrolytes, which have a significant impact on muscle function, various, divergent changes in the levels of K^+^ and Ca^2+^ were observed (Millet et al., 2011), as well as an inverse correlation between the concentration of Na^+^ and CK measured after the run (Magrini et al., 2017). A quite common ailment such as exercise-associated hyponatremia (EAH) is also related to CK levels. During a 24-h run, the occurrence of EAH precedes an increase in CK, and possibly even increases it (Cairns and Hew, 2016).

Another marker of muscle health is myoglobin. Its level increases with the ultramarathon distance (Millet et al., 2011; Kupchak et al., 2014; Jastrzȩbski et al., 2015a), where in the case of running a 100-km distance, the upper norm was exceeded by a factor three-times on average. However, similar to CK, levels of this marker also show high inter-individual variability (Jastrzȩbski et al., 2015a). The measurements show that a 5-day recovery is sufficient to stabilize myoglobin levels within a range approaching baseline levels (Millet et al., 2011).

Myostatin is a negative regulator of muscle growth, and its concentration depends on the intensity and duration of exercise. Tested among the participants of the Spartathlon (246km), myostatin showed a significant increase (Kerschan-Schindl et al., 2015). At the same time, the concentration of follistatin, a substance that prevents myostatin from attaching to the receptor, increased almost 4-fold immediately after completing the run, compared to the baseline values (Kerschan-Schindl et al., 2015).

The presence of fast forms of myosin (FM) in the blood serum is believed to be specific to fast skeletal muscles, whereas the slow forms of myosin (SM) may indicate damage to both skeletal muscles and, in part, cardiomyocytes. During an 85-km-long mountain run, changes were observed in the form of increased SM concentrations. Serum CK and serum SM levels are highly correlated with each other, peaking with a 1-day difference. In the case of FM, no significant changes were observed (Carmona et al., 2015).

An increase in aspartate aminotransferase (AST) is correlated with an increase in CK and myoglobin according to one study (Kupchak et al., 2014). Aminotransferases, AST, and alanine aminotransferase (ALT), do not show a uniform increase. The AST level is much more elevated than the ALT level and, combined with a lack of gamma-glutamyl transpeptidase increase, suggests that changes in the concentration of these parameters more likely result from muscle damage than liver damage caused by prolonged exercise (Skenderi et al., 2006; Kupchak et al., 2014; Jastrzȩbski et al., 2015b; Son et al., 2015).

Several studies have shown that the level of lactate dehydrogenase (LDH) is also associated with running ultramarathon distances and the related damage to muscle fibers. This marker rises with increasing distance traveled (Skenderi et al., 2006; Millet et al., 2011; Jastrzȩbski et al., 2015b) and its level returns to baseline after more than 9days of recovery (Millet et al., 2011). After traveling 75km and during regeneration after 100km, an increase in the so-called liver tests and LDH it was higher for the older age group in one study (Jastrzȩbski et al., 2015b; Tables 1 and 2).

**Table 1:** Muscle markers after participating in ultramarathon.

CK	↑
CK-MM	↑
CK-MB	Smaller increase than CK-MM, but noticeable ↑
cTnI	Much smaller increase than in case of acute coronary syndromes, but noticeable ↑
Myoglobin	↑
Myostatin	↑
Follistatin	↑
SM	↑
FM	-
AST	↑
ALT	↑
LDH	↑

CK, creatine kinase; CK-MM, creatine kinase isoenzyme MM; CK-MB, creatine kinase isoenzyme MB; cTnI, cardiac troponin I; SM, slow myosin isoform; FM, fast myosin isoform; AST, aspartate transaminase; ALT, alanine transaminase; and LDH, lactate dehydrogenase.

**Table 2:** Factors affecting CK increase after an ultramarathon.

Increase of the distance	↑
Second half of the distance	↑
Age	No significant impact
Sex	No significant impact
Training history	No significant impact
Experience in ultrarunning	No significant impact
Highest week running distance	No significant impact
Race finish time	Controversial factor
EAH occurrence	Possible ↑ factor

EAH, exercise-associated hyponatremia.

### Bones and Cartilage Parts

Compared to the markers for damage to muscle fibers, fluctuations in markers for damage to cartilage tissues of the locomotor system are less noticeable in laboratory tests (Kim et al., 2009). Besides, researchers studying ultramarathoners focus their attention on cartilage less frequently than muscle tissues. The amounts of training of ultrarunners may have negative effects, because of altered hormone responses overriding the load-induced impact on bone formation (Chilibeck et al., 1995).

With regard to the markers of bone and cartilage components, Dickkopf 1, an antagonist of the Wnt/β-catenin pathway activating osteoblasts, decreased slightly after running. The same was true of N-terminal propeptide of type I procollagen, a marker of bone formation (Kerschan-Schindl et al., 2015). On the other hand, the level of C-terminal telopeptide of type I collagen, a bone resorption marker, increased as a result of participation in ultramarathons (Kerschan-Schindl et al., 2009, 2015). A decrease in the level of osteocalcin was also observed, the factor indicating the activity of osteoblasts, immediately after the run. These changes were no longer observed 3days later (Mouzopoulos et al., 2007; Kerschan-Schindl et al., 2009). Receptor activator of nuclear factor kappa-β ligand (RANKL) and osteoprotegerin (OPG) proteins showed an increase in measurement after ultramarathon completion. After 3days, the RANKL level increased further and the OPG decreased slightly, but still these markers did not reach the initial values (Kerschan-Schindl et al., 2009).

Taken together, all the markers tested indicate a change in the balance of bone metabolism. A temporary suppression of osteoblasts occurs, but this catabolic effect is short-lived and presumably related to the repair of microdamages associated with strenuous exercise, and over time bone growth resumes (Mouzopoulos et al., 2007; Kerschan-Schindl et al., 2009, 2015).

Immediately after completing a 245-km run, concentrations of bone formation markers were measured in participants. The C-terminal propeptide type I procollagen decreased and then returned to baseline levels within 3days. Additionally, the level of the bone alkaline phosphatase (b-ALP) isoform decreased and then increased on the following day, and on the 5th day it reached pre-run levels (Mouzopoulos et al., 2007). A decrease in hydroxyproline concentration was observed after the run, which then reversed, returning to the values before the run, and then increased until the 5th day after the end of the run. However, because changes in calcium levels did not occur, the origin of these fluctuations is thought to be associated with sources other than bone, such as tendons and skin (Mouzopoulos et al., 2007).

Within the same study, the concentration of cortisol increased by up to 50% compared to the value before the run and quickly returned to normal, on the 1st day of regeneration. A negative correlation has been observed between the level of this hormone, parathyroid hormone (PTH), b-ALP levels, and osteocalcin (Mouzopoulos et al., 2007). An increase in cortisol levels is believed to escalate bone resorption (Mouzopoulos et al., 2007; Kupchak et al., 2014). Another important hormone in this regard is PTH, its increased levels after the run cause the suppression of osteoblasts. Normalization of PTH levels takes place until the 5th day of regeneration after the run (Mouzopoulos et al., 2007). Cartilage oligomeric matrix protein (COMP) is regarded as a sensitive and early indicator of cartilage collagen damage. An increase in this biomarker during running, especially in the second half of an ultramarathon, is now considered an indicator of damage to or hypertrophy of cartilaginous parts (Kim et al., 2007). During a 200-km run, a 1.9-fold increase in COMP in runners’ serum was observed compared to pre-run concentration. This parameter returned to baseline on the 6th day of recovery after the run (Kim et al., 2009; Table 3).

**Table 3:** Bone and cartilage markers after an ultramarathon.

Dkk-1	↓
P1NP	↓
CTX	↑
Osteocalcin	↓
RANKL	↑
OPG	↑
PIPC	↓
b-ALP	Firstly ↓, afterwards ↑
Hydroxyproline	Firstly ↓, afterwards ↑
Cortisol	↑
PTH	↑
COMP	↑

Dkk-1, Dickkopf-related protein 1; P1NP, N-terminal propeptide of type I procollagen; CTX, C-terminal telopeptide of type I collagen; RANKL, receptor activator for nuclear factor κB ligand; OPG, osteoprotegerin; PIPC, C-terminal propepide of type I procollagen; b-ALP, bone alkaline phosphatase; PTH, parathyroid hormone; and COMP, cartilage oligomeric matrix protein.

### Inflammation

In addition to the above changes, an increase in the markers of acute inflammation due to tissue damage, such as interleukin 6 (IL-6), has also been noted (Fallon, 2001; Nieman et al., 2005; Skenderi et al., 2006; Kim et al., 2007; Jastrzȩbski et al., 2015b; Son et al., 2015; Longman et al., 2018; Skottrup et al., 2020). Tumor necrosis factor α (TNF-α) levels remain unchanged, possibly because they are influenced by higher intensity exercise and inhibited by IL-6 (Kim et al., 2007). Many other cytokines can react to ultradistance running. During the Western States Endurance Run (WSER, 160km), measurements of individual interleukins, chemokines, and growth factors were performed. An increase in the following parameters was demonstrated; levels of increase of a given cytokine in relation to the values obtained before the run are given in brackets: IL-6 (125x), IL-10 (24x), granulocyte colony-stimulating factor (G-CSF, 12x), IL-1RA (7x), IL-8 (5x), monocyte chemoattractant protein 1 (MCP-1, 3x), and macrophage inflammatory protein (MIP)-1β (1.2x; Nieman et al., 2005).

The situation is similar during multi-stage ultramarathons. Acute phase proteins were measured during a 6-day competition. Of the 11 subjects, a typical acute inflammatory response was observed for parameters such as iron, transferrin saturation, C-reactive protein, erythrocyte sedimentation rate (ESR), and haptoglobin. On the other hand, five subsequent indicators – transferrin, albumin, α1-antitrypsin, complement components (C3 and C4) – did not show such changes (Fallon, 2001). The data suggest the occurrence of an acute phase reaction in the above-mentioned cases, although, changes in specific parameters are only partially characteristic of inflammation. These discrepancies can be explained to some extent by other effort-related changes (Fallon, 2001; Table 4).

**Table 4:** Inflammation markers after an ultramarathon.

IL-6	↑
TNF-α	-
IL-10	↑
G-CSF	↑
IL-1RA	↑
IL-8	↑
MCP-1	↑
MIP-1β	↑
hs-CRP	↑
Iron	↓
Transferrin saturation	↓
ESR	↑
Haptoglobin	↑
Transferrin	-
Albumin	-
α_1_-antitrypsin	-
Complement components (C3 and C4)	-

IL-6, interleukin 6; TNF-α, tumor necrosis factor α; IL-10, interleukin 10; G-CSF, granulocyte colony-stimulating factor; IL-1RA, interleukin-1 receptor antagonist; IL-8, interleukin 8; MCP-1, monocyte chemotactic protein 1; MIP-1β, macrophage inflammatory protein 1β; hs-CRP, high-sensitivity C-reactive protein; and ESR, erythrocyte sedimentation rate.

The observed changes in the concentration of biomarkers of the musculoskeletal system are relatively short-lived and largely dependent on the length of the distance traveled as well as many less-common factors.

## Muscle Adaptation

Over time, in long-distance runners, the skeletal muscles undergo a number of adaptations. Type I and II fibers of the lower limb muscles of high-performance long-distance runners are characterized by a higher contraction rate compared to people with low activity (Crenshaw et al., 1991). Endurance running promotes the reconstruction of the mechanics of contraction of individual muscle fibers, thus optimizing their function (Harber and Trappe, 2008). The composition is dominated by type I fibers, characterized by slow-twitch but high-strength (Crenshaw et al., 1991; Harber and Trappe, 2008).

Selective hypertrophy of type I fibers can occur (63.0–78.3% of the total number of fibers; Crenshaw et al., 1991). For this reason, the SM marker seems to better reflect the degree of muscle damage during ultramarathons. To further increase specificity, cTnI was compared to SM, which revealed an increase in cTnI due to damage to cardiomyocytes. However, the increase in cTnI was so low that the SM index could be regarded as largely specific for type I myocyte damage (Carmona et al., 2015).

Noticeable changes in muscle structure also take place on a microscopic scale. In a study of the myocytes of ultramarathon runners, interesting cellular changes were found on the day following the 100-mile distance (Crenshaw et al., 1991). Centrally located muscle fibers were more densely surrounded by capillaries than peripheral fibers. Type I fibers were abundant and richly vascularized. In addition, both fiber types I and II had a 20% larger diameter compared to recreational runners (Harber and Trappe, 2008). A linear relationship was also noted between the fiber size and the associated vascular density, which actually determines the diffusion distance. The numerous mitochondria were especially densely located in the vicinity of the sarcolemma and near the vessels. On the whole, these changes are considered an adaptation to endurance effort (Crenshaw et al., 1991).

When half-marathon runners, marathon runners, and ultrarunners are compared, all runners have similar muscle mass, but the latter have a lower percentage of body fat (Knechtle et al., 2012b). Compared to marathon runners, top ultrarunners have more muscle mass in the lower limbs. This does not allow ultrarunners to develop the speed of runners in shorter distances, but greater muscle mass in the lower limbs positively influences endurance, which is important in this discipline (Millet et al., 2012). Other studies have shown that skeletal muscle mass is not correlated with the completion time of ultramarathon runners (Knechtle et al., 2012b).

The general trend is that, with age, muscle mass decreases, and so does VO_2_ max. This results in poorer performance in endurance races such as ultramarathons (Trappe et al., 1996; Knechtle et al., 2012b). However, aerobic capacity does not decrease in response to exercise during ultramarathons (Efficacy et al., 2014). Loss of muscle fibers begins around the age of 50 and occurs largely in the lower extremities (Faulkner et al., 2007). This process occurs regardless of the degree of training, and while still physically active, this decline can be partially prevented (Trappe et al., 1996). Among ultramarathon runners, the type of training has not been shown to affect the percentage of muscle mass, but it has been shown to affect the percentage of fat mass (Knechtle et al., 2012b).

In studies on the influence of running a 161-km ultramarathon, a decrease in testosterone in men was shown. This change is thought to be an adaptation to regular intense exercise (Hackney et al., 2003). For ultrarunners, the reduced level of testosterone limits protein synthesis and thus the development of muscle mass, which reduces unnecessary weight and the associated energy expenditure during ultramarathons. The constantly maintained level of this hormone below the reference values may lead to a decrease in the intensity of spermatogenesis as well as a decrease in bone mineral density (Hackney, 1996; Fournier et al., 1997; Kupchak et al., 2014; Longman et al., 2018).

## Injuries Among Ultramarathon Runners

As for any physical activity, characteristic types of health problems can also be distinguished for running distances above a marathon. During the run, the following factors influence the result to a large extent: minimization of muscle damage and gastrointestinal symptoms, as well as internal motivation (Millet et al., 2012). The degree of muscle-tendon and osteoarticular damage is very important. On the other hand, in order to avoid gastric discomfort, runners find it essential to eat foods that have already been tried by other athletes in the past while running (Costa et al., 2019). Although, ultramarathon runners can boast of above-average overall health, they are commonly affected by injuries. As many as 64% of medical visits by ultrarunners are related to injuries. Ultrarunners utilize the help of several medical care specialists mainly as: physiotherapist, 47.6%; chiropractor, 24%; podiatrist, 12.5% (Hoffman and Krishnan, 2014). The frequency of injuries is influenced by such factors as regularity of training sessions and distances covered during them, age, running experience, type of ground, intensity of and practicing activities that diversify training (Hoffman, 2016). Regardless of the source, the evidence is unambiguous – injuries are a serious problem among ultramarathoners. The majority, as many as 77% of respondents, report the occurrence of an injury in the last year, with 64.6% specifying injuries that caused a minimum 1-day break in training (Hoffman and Krishnan, 2014). In another study, this percentage is 52.2% (Hoffman and Fogard, 2011).

Many similarities exist between marathon and ultramarathon runners. However, noticeable differences occur between these two types of competitors related to the different specificity of the races themselves and common injuries and dysfunctions affecting participants of both distances (Khodaee and Ansari, 2012; Lopes et al., 2012; Millet et al., 2012; Hoffman, 2016). Hence, the results of research conducted on marathon runners cannot be fully translated to ultramarathon runners. A comparison between marathon runners and ultrarunners with regard to types of injuries shows that the former tend to undergo acute tendon pathologies more often, whereas the latter tend to suffer from chronic tendon injuries more often (Krabak et al., 2011, 2014).

The prevalence of musculoskeletal injuries during competition is unquestionable. In a study of injuries occurring during multi-day off-road ultramarathons, musculoskeletal injuries were the second most common (19%; Lopes et al., 2012; 18.2%; Schwabe et al., 2014) after skin injuries (70%; Krabak et al., 2011, 2014; Khodaee and Ansari, 2012; Vernillo et al., 2016). The studies distinguishing single-stage and multi-stage ultramarathons showed that in both cases the vast majority of injuries (locomotor system, 93%; skin, 99%) were classified as not serious enough to prevent runners from continuing (Krabak et al., 2011, 2014; Vernillo et al., 2016). The third most common was tendinitis (11.3%). In addition, sprains, tears, and bursitis were less frequent (Krabak et al., 2011). Broadly speaking, running injuries got during the competition lead to only 7.9% of dropouts, and injuries which were present before performance to 7.2% (Hoffman and Fogard, 2011).

Injuries to the motor system of ultramarathon runners are most often located in the lower limbs (92.6%; Krabak et al., 2011; Khodaee and Ansari, 2012; Hoffman and Krishnan, 2014; Vernillo et al., 2016). During the multi-stage ultramarathons, in terms of frequency of occurrence, the foot (73.7%), the shin (8.6%), the ankle (4.9%), and the knee (3.5%) show a descending pattern (Krabak et al., 2011). In contrast, one study found the knee to have the highest incidence of injuries (24%; Hoffman and Krishnan, 2014). Taking into account the different types of injuries, tendinitis is the most common, occurring as damage to the Achilles tendon (incidence 2–18.5%; Hoffman and Fogard, 2011; Lopes et al., 2012; Vernillo et al., 2016) patellofemoral pain syndrome (7.4–15.6%; Lopes et al., 2012; Hoffman and Krishnan, 2014); and extensor tendonitis (Bishop and Fallon, 1999; see Table 5 for the full spectrum of injuries). In the case of multi-day ultramarathons, the 3rd to 4th day is observed to be a “tipping point,” where injuries are most often reported by runners (Krabak et al., 2011, 2014). The time required to complete a multi-day run does not affect the frequency of injuries, generally counting and distinguishing injuries to the musculoskeletal system (Krabak et al., 2011).

**Table 5:** Incidence of exercise-related injuries among ultrarunners.

Study	Hoffman and Krishnan, 2014	Vernillo et al., 2016	Hoffman and Fogard, 2011	Lopes et al., 2012
*n*	1,212	77	500	126
Injury type and/or location	Incidence of injuries (% of affected runners)			
Fractures not involving the extremities	1			
Upper extremity injuries (including fractures)	1.4			
Back injuries	12.4		4.2	
Iliotibial band issue	15.8		7.3	4.7
Hip flexor strain	8.7	14.3	4.2	1.0–4.7
Hamstring strain	11.8		11.1	
Stress fracture involving femur or hip	0.5		1.1	
Other leg, pelvis, or hip issues	3.7			
Knee issues	24	14.3	19.9	
Calf strain	13.1		8.8	
Achilles tendinitis or tear	9.2	7.1	11.5	2.0–18.5
Stress fracture involving tibia or fibula	1.9		3.8	
Other lower leg injuries	1.5			
Ankle sprain	10.8	28.6	9.2	5.1
Plantar fasciitis	10.6	28.6	9.6	
Stress fracture involving foot	3.4		4.6	
Morton’s neuroma	3.1		1.9	
Metatarsalgia	3.1		1.5	
Great toe metatarsal phalangeal joint pain (bunion)	2.5		0.8	
Other foot and ankle injuries	4.5			
Skin wounds, blisters, and infections	1.5			
Neck/cervical spine strain		7.1		
Patellofemoral syndrome				7.4–15.6
Medial tibial stress syndrome				7.8–11.1
Patellar tendinopathy				6.3–18.5
Trochanteric bursitis				3.0–3.1
Psoas bursitis				11.1

Because of mountain specificity in a large number of ultramarathons, characteristic groups of muscles subject to special loads were observed (Millet et al., 2011; Hoffman et al., 2012; Degache et al., 2014). A large total number of surges, both ascents and descents, increases the involvement of the plantar flexors of the ankle joint in relation to the dorsal flexors (Degache et al., 2014).

Among the complaints affecting soft tissues within the locomotor system are exercise-associated muscle cramps (EAMCs). They are a very common affliction of ultramarathon runners and concern 19–26.2% of competitors (Schwellnus et al., 2011; Khodaee and Ansari, 2012; Schwabe et al., 2014; Vernillo et al., 2016). EAMCs account for 5% of all reasons for quitting the competition (Hoffman and Fogard, 2011; Khodaee and Ansari, 2012). Factors predisposing runners to EAMCs include: past history of EAMCs, faster covering of the first part of the distance (in the 28/56km study), history of a fall during an ultramarathon (in this case, the probable share of accompanying neuromuscular disorders increases), prolonged duration of activity (especially during the last ¼ of the distance), and greater pain in the quadricep muscles of the thigh after running related to eccentric contractions (Schwellnus et al., 2011). In addition, putative risk factors include increased training intensity in the 3days prior to the start and longer training units in general, more stretching exercises during training, and greater concentration of CK before the race (which may suggest greater muscle damage among the EAMC risk group; Schwellnus et al., 2011). More experienced ultramarathoners are less likely to experience problems such as muscle cramps. Male gender is a likely risk factor for EAMCs (Schwabe et al., 2014).

Muscle strains are a common problem among ultramarathoners, affecting 41% of runners (Hoffman and Stuempfle, 2016), and are also mentioned by 5% of runners as the reason for their resignation from competitions (Hoffman and Fogard, 2011). This problem most often affects the muscles most active during the run, the lower leg (13.1%), the quadriceps (11.8%), and the posterior group of the thigh (Hoffman and Krishnan, 2014; Hoffman and Stuempfle, 2016). Muscle tears are particularly common in ultramarathon runners who have suffered them in the past (Schwellnus et al., 2011; Hoffman and Stuempfle, 2016) and in runners with severe damage (elevated CK values) and with increased urea nitrogen in blood (Hoffman and Stuempfle, 2016).

Only in some studies has there been a difference between the incidence of injuries and gender. Women are injured more often than men in general, but the incidence of the different groups of injuries is the same (Krabak et al., 2011; Hoffman and Krishnan, 2014; Schwabe et al., 2014). The incidence of stress fractures is a certain exception, given that women are predisposed (Hoffman and Krishnan, 2014). Anatomy as a group, ultrarunners suffer less fatigue fractures than runners of shorter distances. This difference is due in part to the fact that ultramarathon runners spend a significant fraction of their distances on hard ground, such as asphalt (Hoffman and Krishnan, 2014; Hoffman, 2016). However, the location of the injury itself differs depending on the terrain. In the case of ultramarathon runners, this type of injury affects the foot more often, which may result from the greater stress on this area when running on less level terrain and length of the distance traveled (Hoffman and Krishnan, 2014). In addition, ultramarathon runners running on soft trails more often have problems in the ankle joints, whereas ultramarathon runners running on hard ground, such as the street, are more prone to injury to the knee joints (Bishop and Fallon, 1999).

When analyzing a group of runners who suffered injuries over the last year, the individuals were statistically younger, less experienced, relatively less focused on running, spent more of their training units on high-intensity activities, and more regularly included resistance training (Krabak et al., 2011; Hoffman and Krishnan, 2014; Schwabe et al., 2014; Knechtle and Nikolaidis, 2018). Older runners have some advantage: a 10-year age difference is related to a decrease in the overall incidence of injuries by half, and of injuries within the locomotor system by 0.2 (Krabak et al., 2011). These advantages may be explained as adaptations of the ultramarathon runner’s body resulting from years of practicing sports, training experience, and/or favorable genetic features that allow for many years of running (Hoffman and Krishnan, 2014).

Virtually all ultrarunners report pain after competing that is distinguished into two types, muscle soreness related to fatigue and specific injuries (Khodaee and Ansari, 2012). DOMS develops more often due to eccentric muscle contractions (Visconti et al., 2015). When DOMS affects the lower limbs, this condition is especially severe for runners, as it causes a loss of maximum contractile force and a marked discomfort. After completing the run, more than 90% of runners complain of pain in the lower limbs, with 60% indicating the calves, 23% indicating the thighs, and 8% indicating the knee area (Visconti et al., 2015). The factors correlated with the level of pain experienced include age of the competitor, experience in running ultramarathons, and CK concentration immediately after the run. Statistically, runners with CK concentrations of ≥28,000UL^−1^ reported pain 1.5 times stronger than those with CK concentrations below 10,000UL^−1^ (Hoffman et al., 2017).

One of the reasons suggested to explain how ultramarathon runners are able to cover longer distances compared to other runners is that they have a higher pain tolerance (Freund et al., 2013). The research shown that running the ultramarathon can cause partial analgetic effect induced by the effort, even up to 30min. Improved pain tolerance lasted the longest in the fastest runners, and was completely absent in the slower runners. Because slower runners completed the final stage at lower speeds, they may not have experienced the same effect (Hoffman et al., 2007).

Due to the prolonged effort associated with long-distance running, neuromuscular transmission is weakened. The consequence of this condition is a reduction in the maximum arbitrary muscle activation. The loss of maximal voluntary contraction (MVC, a measure of muscle strength) was determined noticeable for muscles particularly involved during a ultramarathon mountain run (Millet, 2011; Millet et al., 2011). In case of electromyography (EMG) the changes last longer (Millet et al., 2011). The decreased value of the parameters is related not only to biochemical changes occurring in the central nervous system, but also to peripheral entities involved, which induce neuromuscular weakness. This can be explained as the body’s defense mechanism (Millet, 2011; Millet et al., 2011). The occurrence of the above-mentioned changes in neuromuscular transmission during single and multi-stage ultramarathons was also compared. Central nervous system changes are more noticeable in single-stage competitions, whereas peripheral nervous system changes last longer after multi-stage runs due to their greater intensity (Besson et al., 2020).

Changes in the biomechanics of running during an ultramarathon are associated with adopting a safer running technique – the movements become smoother, the energy expenditure incurred for vertical movements decreases and the frequency of steps increases (Degache et al., 2016; Thompson, 2017). Significant modifications were observed between the starting point and the middle stage of an ultramarathon. At a later stage of the course, no changes take place (Degache et al., 2016). Modifications are aimed both at reducing the sensation of pain and minimizing damage to the musculoskeletal system, especially damage caused by eccentric contractions expressed during downhills (Millet, 2011; Degache et al., 2016). The greater the variation in the length of the running stride, the greater the risk of injuries (Millet et al., 2012). Importantly, the purpose of these changes is not seen as a reduction in energy costs (Millet, 2011).

## Factors Influencing Recovery and the Risk of Injury

Although, a great deal of research has been conducted on how to prevent injuries to the locomotor system among runners, most studies are based on marathon runners, which cannot be clearly translated to ultramarathon runners. Thus, a clear gap exists in the research examining ultramarathons (Krabak et al., 2013).

Taking into account the experience of ultramarathon runners, attention was drawn to the fact that in order to minimize the risk of injuries, the number of starts in competitions during the season should be limited, gradually increasing the mileage during training and maintaining a balanced pace during the competition (Krabak et al., 2011). Competitors running at a statistically average pace have a smaller the risk of injuries in relation to those running faster or slower (Schwabe et al., 2014). Paying attention to the gradual increase of loads during training, and practicing activities outside of competition that are particularly demanding during competition (e.g., downhill runs), can help minimize the harmful effects of concentric and eccentric contractions on the muscles, and thus increase the resistance to this type of load (Efficacy et al., 2014). When training for a given competition, runners benefit from exercising in conditions similar to those of the target race, in terms of the type of surface, terrain, temperature, and climate (Krabak et al., 2013). A gear supply can also be a potentially important factor to help the runner finish the run successfully. Therefore, any equipment should be tested before starting a race (Krabak et al., 2013).

Although, compression socks are promoted for long-distance runners, research has shown that they provide no benefit: they do not have a positive effect on the structure and volume of the muscles of the lower limbs, nor on the results obtained. Paradoxically, runners from the research group that tested compression socks reported stronger pain after running an ultramarathon (56km). The pain was probably due to the discomfort associated with wearing such compression elements (Geldenhuys et al., 2019).

Another factor contributing to the increase in the incidence of injuries is sleep deprivation, which is common among ultramarathon runners who cover multi-day distances (Degache et al., 2016; Larson et al., 2016; Martin et al., 2018). Research shows that sleep deprivation, in combination with other factors related to the exertion of running, impairs the control of body posture by reducing alertness, executive functions, and sensorimotor integration (Degache et al., 2014). Combined with the fact that ultramarathons are often organized in areas with difficult, uneven ground (Krabak et al., 2013, 2014; Carmona et al., 2015; Degache et al., 2016; Hoffman, 2016; Larson et al., 2016) sleep deprivation results in an increased risk of falls and injuries.

A significant difference in a short tandem-repeat polymorphism (STRP rs71746744) of the gene for the α1 chain of collagen V (COL5A1) was demonstrated among runners. Depending on the genomic variant, the competitors showed differences in flexibility and speed of finishing the run. People with the −/− genotype showed much greater flexibility, but were slower than athletes with the AGGG/AGGG and −/AGGG genotypes (Abrahams et al., 2014). Furthermore, the rs71746744 AGGG/AGGG genotype (fast, but less flexible runners) occurs much more often in people with chronic Achilles tendonitis (Abrahams et al., 2014). The above genotypic system may be one of many possible genetic adaptations to the discipline of ultramarathon running that constitute an interesting area of the future research.

Although, the use of nonsteroidal anti-inflammatory drugs (NSAIDs) before and during a run is common practice among athletes (Nieman et al., 2005; Hoffman and Fogard, 2011; Francisco, 2013; Hoffman et al., 2016; Larson et al., 2016) the drugs do not have a positive effect on the perception of muscle pain (Nieman et al., 2005; Francisco, 2013; Hoffman et al., 2016). Some researchers even indicate an increased feeling of DOMS in the first the day after the competition with the use of NSAIDs (Francisco, 2013).

Short-term amino acid supplementation, lasting between 12 and 13h, before and during the run, did not affect the degree of muscle damage, subjective pain sensations, time to finish the run, and impaired kidney function. These results may be related to the amount of time the supplements were used. Although, the presumption is that positive effects can be expected with prolonged use, this relationship should be subjected to long-term research (Knechtle et al., 2011, 2012a; Costa et al., 2019).

Still not fully recognized, vitamin D_3_ supplementation can be a significant element of the diet affecting an athlete’s performance and regeneration. According to research, vitamin D_3_ has a significant, beneficial effect on iron levels. In this way, it prevents the iron deficiency anemia that often affects athletes (Kasprowicz et al., 2020). In addition, a negative correlation has been noted regarding vitamin D_3_ levels and markers of muscle damage. This may have a positive effect on regeneration after ultramarathon running, but no detailed recommendations are known for effective vitamin D_3_ supplementation among competitors in this discipline (Zebrowska et al., 2020).

The concentration of CK after running, which is closely related to the degree of muscle damage, is considered the principal and consistent factor related to the rate of recovery (Hoffman et al., 2017). Above all, proper training has an impact on lower CK release from muscle fibers due to less damage to myocytes, and this is also related to a faster recovery rate after the run. The broadly understood adaptations resulting from the constant subjecting of the body to endurance effort are also helpful in this regard (Millet et al., 2012; Hoffman et al., 2017).

In order to accelerate regeneration, various forms of massage or pressure are used. These techniques are aimed primarily at increasing the return of lymph and blood from the lower extremities. The use of a 20-min session of specialized massage using the “effleurage” technique among ultramarathon runners was investigated, focusing on the DOMS reported after the run. Runners reported a decrease in pain sensation by 3.6 (±2.1) points on a scale of 1–10 after the procedure (Visconti et al., 2015). In another study, immediately after running an ultramarathon (161km), a 20-min massage session and sequential intermittent pneumatic compression of the lower limbs were conducted. Subjective, immediate positive effects on muscle soreness in general and lower extremity muscles specifically were reported (Hoffman et al., 2016).

In the case of long-distance runs, an important issue is the shape and direction of the track on which the competitors move. A track length of 2,000–2,500m is definitely preferred by runners who participate in such competitions. Moreover, the track should have a regular shape, without sharp angles, and the direction of the run should be changed at regular intervals. This is justified in practice, because overload changes of the right side, especially the knee, were observed in a 24-h race on a rectangular loop, only in the clockwise direction. Thus, failing to alter the direction of the run is a risk factor for the occurrence of injuries among runners in this type of competition (Gajda et al., 2020).

## Summary

Acute locomotor system changes in runners as assessed by locomotor biomarkers are reversible, can be asymptomatic or painful, and often are reversible within 1week of the competition. Injuries suffered by runners largely allow them to finish the competition and are usually overlooked. Careful regeneration, including regular massage and the use of supporting techniques, allow for faster convalescence. Given that ultramarathon races are becoming more and more popular, this publication is mean to serve as a source of knowledge for people associated with this discipline.

## Limitations of the Study

Due to the small number of studies conducted on groups of ultramarathoners, the current comparisons include ultramarathoners covering various distances, in different conditions. Each ultramarathon competition is specific, where not only distance is the factor, but also the presence of elevation gains or climate-related issues. At the current level of development of this discipline, the distances vary, resulting in few studies that compare runners only for a specific mileage.

## Author Contributions

AP: conceptualization and visualization. AP and ZW: writing—original draft preparation and writing—review and editing. All authors contributed to the article and approved the submitted version.

## Conflict of Interest

The authors declare that the research was conducted in the absence of any commercial or financial relationships that could be construed as a potential conflict of interest.

## Publisher’s Note

All claims expressed in this article are solely those of the authors and do not necessarily represent those of their affiliated organizations, or those of the publisher, the editors and the reviewers. Any product that may be evaluated in this article, or claim that may be made by its manufacturer, is not guaranteed or endorsed by the publisher.
